# Metabolic cost adaptations during training with a soft exosuit assisting the hip joint

**DOI:** 10.1038/s41598-019-45914-5

**Published:** 2019-07-05

**Authors:** Fausto A. Panizzolo, Gregory M. Freisinger, Nikos Karavas, Asa M. Eckert-Erdheim, Christopher Siviy, Andrew Long, Rebecca A. Zifchock, Michael E. LaFiandra, Conor J. Walsh

**Affiliations:** 1 John A. Paulson School of Engineering and Applied Sciences, Harvard University, 29 Oxford Street, Cambridge, MA 02138 USA; 2 Wyss Institute for Biologically Inspired Engineering at Harvard, 3 Blackfan Circle, Boston, MA 02115 USA; 3 Department of Civil and Mechanical Engineering, United States Military Academy, 752 Thayer Road, West Point, NY 10996 USA; 4 United States Army Research Laboratory, Aberdeen Proving Ground, 4727 Deer Creek Loop, MD 21005 Maryland, USA; 5 75th Innovation Command, United States Army, 10949 Aerospace Ave, Houston, TX 77034 USA

**Keywords:** Musculoskeletal system, Engineering, Biotechnology

## Abstract

Different adaptation rates have been reported in studies involving ankle exoskeletons designed to reduce the metabolic cost of their wearers. This work aimed to investigate energetic adaptations occurring over multiple training sessions, while walking with a soft exosuit assisting the hip joint. The participants attended five training sessions within 20 days. They walked carrying a load of 20.4 kg for 20 minutes with the exosuit powered and five minutes with the exosuit unpowered. Percentage change in net metabolic cost between the powered and unpowered conditions improved across sessions from −6.2 ± 3.9% (session one) to −10.3 ± 4.7% (session five), indicating a significant effect associated with training. The percentage change at session three (−10.5 ± 4.5%) was similar to the percentage change at session five, indicating that two 20-minute sessions may be sufficient for users to fully adapt and maximize the metabolic benefit provided by the exoskeleton. Retention was also tested measuring the metabolic reduction five months after the last training session. The percent change in metabolic cost during this session (−10.1 ± 3.2%) was similar to the last training session, indicating that the adaptations resulting in reduced metabolic cost are preserved. These outcomes are relevant when evaluating exoskeletons’ performance on naïve users, with a specific focus on hip extension assistance.

## Introduction

Lower limb exoskeletons have been proposed as an effective solution for augmenting human walking in individuals with normal gait^1–4^. A common goal for many exoskeletons is to reduce the metabolic cost of walking. In the last ten years, several research laboratories have developed exoskeletons: some fully autonomous^2–5^ and others tethered to external actuators or power supplies^6–9^; these systems reduce the metabolic cost of walking by applying an assistive torque to leg. While these systems have different design characteristics (actuator, control system, total torque applied, lower limb joint assisted, mass, etc.)^10^, the one commonality among all exoskeletons is that individuals must learn how to best utilize the given external assistance. Thus, advancing our understanding of how we adapt to walking with an exoskeleton may allow for enhanced designs and improved performance.

Though many exoskeleton studies include at least one training or familiarization session prior to evaluation^2,4,9,11,12^, only a few present the improvement in metabolic cost that occurs as a result of repeated use or training^7,13^. In one of these studies^7^, the authors used a pneumatically powered ankle exoskeleton to demonstrate that while changes in joint kinematics and muscle activation patterns occurred following the first training session, metabolic cost continued to significantly decrease over three separate sessions on different days. Sawicki and Ferris reported that participants initially had a 7% increase in the metabolic cost of walking on the first day compared to a reduction of 10% on the third day, all in comparison to an unpowered condition. They suggest that changes in net metabolic power may occur more slowly than changes in joint kinematics and muscle activation patterns during adaptation to powered walking^7^. These results conflict with a study by Finley *et al*.^14^ which utilized a split-belt treadmill operating at different belt speeds to investigate adaptation. In this study, participants initially walked with unequal step lengths, over time they took steps of equal length and exhibited a bilateral reduction in EMG. These changes were also associated with a reduction in metabolic cost. This study only included a single testing session, so it is unknown how the metabolic reductions would be impacted with repeated exposure to split-belt treadmill conditions. It is also unknown how motor adaptation varies when inducing asymmetry with varied belt speeds compared to exoskeletons designed to provide torque as beneficially as possible during gait. This fundamental distinction likely provides significantly different changes to the energetic landscape, which may result in altered adaption rates and strategies.

Galle *et al*.^15^ reported the adaptation to an ankle exoskeleton over a single session and found that net metabolic cost decreased ~7% over the course of approximately 14 minutes of walking, while the ankle and knee angles remained relatively stable over that period. The soleus muscle activity was significantly lower from the beginning of adaptation to the minimum metabolic cost time point (18.5+/−5.0 min). However, the gastrocnemius, biceps femoris, vastus lateralis, and tibialis muscle activity were not significantly different. These dramatic changes in metabolic cost over a single session, with variable changes in kinematics and muscle activity, illustrate the potential complexity of metabolic adaptation. These studies emphasize the importance of allowing participants sufficient time to metabolically adapt to the exoskeleton, since shorter training time could prevent researchers and wearers from completely appreciating the full metabolic benefit of new exoskeletons^16^. All previous exoskeleton adaptation research has been focused on utilizing an ankle exoskeleton, however little is known about how this work applies to exoskeleton assisting other lower joints. Each joint provides a different amount of mechanical work during walking gait, with the percentage of total average positive power approximately 42%, 17%, and 41% for the hip, knee and ankle respectively^17^. The neuromuscular control strategy and timing for positive and negative power has also been shown to vary between the hip, knee and ankle joint^17,18^. Therefore different adaptation rates may be possible for exoskeletons designed for the hip versus the ankle.

Understandably, there is little consensus on the minimum amount of training or exposure time required by the user to maximize the metabolic benefit associated to walking with an exoskeleton. The metabolic benefit is likely dependent on (i) the specific controller used^19^, (ii) the physical characteristics of the device and iii) the specific individual being tested^20^. To date, the longest-term exoskeleton training studies^7,13^ reported adaptations across three training sessions. Only a study by Sawicki and Ferris performed pilot testing of a fourth session, in this work the authors did not see any further reduction in metabolic cost in three individuals^7^. There is also a scarcity of research on the long-term retention of metabolic benefits following initial training with an exoskeleton. Maeda *et al*. have shown that participants can retain novel visuomotor mappings during a precision walking task one-year following training^21^, which highlights the robustness of neural changes in the motor sequences associated with walking. Motor learning research in the upper extremity has shown similar retention of certain tasks ranging from 5 months to 1 year following initial adaptation^22–24^, along with structural changes to brain gray matter concentrations^22^. Humans are able to consistently alter walking strategy to optimize for metabolic cost^25,26^, however it is currently unknown if the metabolic benefits due to exoskeleton training are retained after prolonged periods of non-use. Understanding the effects of longer-term training and retention of improved performance are important for the development and the adoption of exoskeletons assisting gait.

The purposes of this study were to: (i) understand the effect of extended training sessions with a soft exosuit assisting the hip, on the changes in metabolic cost and (ii) investigate if metabolic improvements due to training were retained following a five month break of use. We hypothesized that metabolic cost would be significantly reduced with each additional training session. Furthermore, we hypothesized there will be no difference in metabolic cost reduction following a five month break in use. To test these hypotheses we assessed metabolic cost, subjective evaluations, and stride time across five training sessions. During each training session the participant walked for 20 minutes with the exosuit powered, and five minutes with the exosuit unpowered. For data collection and analysis, the 25 total minutes of walking were divided in four segments: *early_pow* (0–2 minutes), *mid_pow* (9–11 minutes), *late_pow* (18–20 minutes) and *unpow* (23–25 minutes). To understand the effect of retention following the initial adaptation process, participants were retested five months after the completion of their last testing session.

## Results

### Metabolic cost

**Figure 1:**
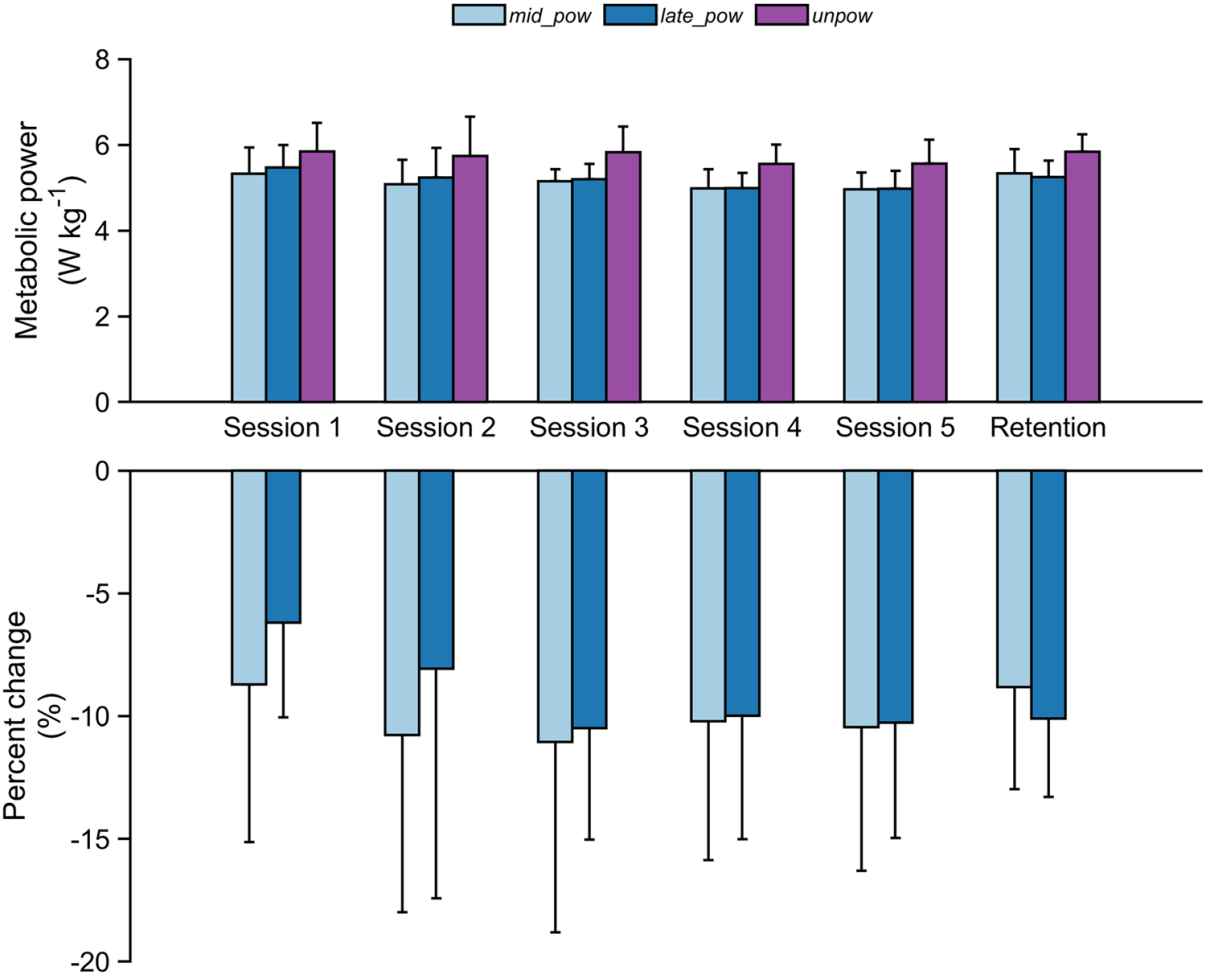
Net metabolic cost exhibited by the participants across the five training sessions (n = 8) and 5-month follow-up of retention (n = 5). Absolute values recorded during walking with the soft exosuit powered at minutes 9–11 (*mid_pow*), at minutes 18–20 (*late_pow*) and unpowered at minutes (*unpow*) and percentage change in metabolic cost for *mid_pow* and *late_pow* with respect to *unpow*. Data are mean ± standard deviation.

Statistical analyses showed a significant within-sessions effect of the net metabolic cost of walking across all the training sessions (Table 1). Average metabolic power during the first two minutes of walking (*early_pow*) was not included in the analysis as this did not provide enough time to reach steady state metabolic energy expenditure^27^. Post-hoc comparisons revealed that net metabolic power was significantly lower at both *mid_pow* and *late_pow* compared to *unpow* in the first testing session (p = 0.002 and p = 0.019, respectively) and across all the testing sessions (Fig. 1 and Table 1). No differences in the net metabolic power were reported between *mid_pow* and *late_pow* across any of the five training sessions (p = 0.470, p = 0.701, p = 0.940, p = 0.997 and p = 0.989, respectively). Statistical analyses also reported a significant across-sessions effect in the reduction of metabolic cost only during *late_pow* across sessions (p = 0.034, Table 1). All net metabolic values within and across sessions are presented in Table 1.

**Table 1:** Net metabolic cost calculated during walking with the soft exosuit powered at minutes 9–11 (*mid_pow*), at minutes 18–20 (*late_pow*) and unpowered at minutes 23–25 (*unpow*) across the five testing sessions.

	*mid_pow*	*late_pow*	*unpow*	p-value
	[W·kg^-1^]	[W·kg^−1^]	[W·kg^−1^]	
Session 1	5.33 ± 0.61	5.47 ± 0.52	5.84 ± 0.67*^§^	0.002
Session 2	5.08 ± 0.57	5.24 ± 0.69	5.74 ± 0.92*^§^	0.01
Session 3	5.15 ± 0.28	5.20 ± 0.36	5.83 ± 0.60*^§^	<0.001
Session 4	4.98 ± 0.45	4.99 ± 0.35	5.56 ± 0.45*^§^	<0.001
Session 5	4.96 ± 0.39	4.98 ± 0.41	5.56 ± 0.56*^§^	<0.001
Across sessions p-value	0.15	0.034	0.584	

^*^Represents statistically significant within session differences with *mid_pow*, ^§^represents statistically significant within session differences with *late_pow* (p < 0.05). Data are means ± standard deviation.

Metabolic cost and the percent change in metabolic cost between powered and unpowered conditions by session is shown in Fig. 1. No significant difference was found in percentage change of *late_pow* vs *unpow* between session one and session two (−6.2 ± 3.9% vs −8.1 ± 9.4%; p = 0.586). A significant difference was identified in percentage change at *late_pow* between session one with respect to session three (−10.5 ± 4.5%; p = 0.031). Similar metabolic reductions were recorded in session four and five; percentage change at *late_pow* vs *unpow* between session one with respect to session four and session five were −10.0 ± 5.0% and −10.3 ± 4.7% (p = 0.054 and p < 0.001 respectively) (Fig. 1). We also explored three additional training sessions in a subset of participants (n = 5). No further reductions in percent change in net metabolic power were reported in those that underwent eight training sessions. Specifically, no significant difference was reported in percentage change between *late_pow* for session five and session eight (−11.4 ± 5.7% vs −12.3 ± 4.6%, respectively; p = 0.772).

Another subset of participants (n = 5) was tested five months after their final training session to assess retention of these metabolic benefits. We found no significant difference in percentage change between *late_pow* recorded during the last training session and *late_pow* recorded during the retention follow up session (−11.1 ± 3.0% vs −10.1 ± 3.2%, respectively; p = 0.474).

### Subjective evaluations

A significant within-session effect was found relative to the rate of perceived exertion for each training session (Table 2). Participants reported lower perceived exertion when walking with the soft exosuit powered (both at *mid_pow* and at *late_pow*) with respect to walking with the soft exosuit unpowered. There were no significant differences in perceived exertion between sessions. The results relative to the subjective evaluations are reported in Table 2.

**Table 2:** Borg scale of perceived exertion during walking with the soft exosuit powered at minutes 9–11 (*mid_pow*), at minutes 18–20 (*late_pow*) and unpowered at minutes 23–25 (*unpow*) across the five testing sessions.

	*mid_pow*	*late_pow*	*unpow*	p-value
Session 1	10.8 ± 0.9	11.5 ± 1.4^*^	12.4 ± 1.1*^§^	<0.001
Session 2	10.3 ± 1.3	11.5 ± 1.4	13.0 ± 1.2*^§^	<0.001
Session 3	10.4 ± 1.3	11.1 ± 1.1	12.3 ± 1.7*^§^	0.007
Session 4	10.5 ± 0.9	11.1 ± 1.2	12.4 ± 1.4*^§^	<0.001
Session 5	10.8 ± 1.0	11.3 ± 1.0	12.3 ± 1.5*^§^	<0.001
Across sessions p-value	0.456	0.494	0.14	

^*^Represents statistically significant within session differences with *mid_pow*, ^§^represents statistically significant within session differences with *late_pow* (p < 0.05). Data are means ± standard deviation.

### Stride time

No across sessions effect was reported in the stride time, however a significant within-sessions effect was identified (Table 3). In particular, stride time increased from the beginning (*early_pow*) to the end (*unpow*) of all training sessions. However, stride time was not significantly different between *late_pow* and *unpow*.

**Table 3:** Stride time (seconds) during walking with the soft exosuit powered at min 0–2 (*early_pow*), min 9–11 (*mid_pow*), at min 18–20 (*late_pow*) and unpowered (*unpow*) across the five testing sessions.

	*early_pow*	*mid_pow*	*late_pow*	*unpow*	p-value
Session 1	0.98 ± 0.04	1.00 ± 0.05	1.00 ± 0.05	1.01 ± 0.05^^^	0.0017
Session 2	0.98 ± 0.04	1.00 ± 0.05	1.01 ± 0.06*	1.02 ± 0.04*	<0.001
Session 3	0.99 ± 0.05	1.01 ± 0.05	1.01 ± 0.05	1.02 ± 0.04*	0.026
Session 4	0.98 ± 0.04	1.01 ± 0.04^*^	1.01 ± 0.04^*^	1.01 ± 0.04^*^	0.002
Session 5	0.99 ± 0.04	1.01 ± 0.04^*^	1.01 ± 0.05^*^	1.01 ± 0.04^*^	0.008

^*^Represents statistically significant within session differences with *early_pow* (p < 0.05). Data are means ± standard deviation.

## Discussion

This study investigated the adaptations in metabolic cost that occur while walking with a hip assistive device across a relatively high number of sessions. We also examined the retention of any metabolic benefits to training, following five months of non-use. While this study was focused on the effects of training, the metabolic cost of walking with the soft exosuit powered was significantly lower than that with the soft exosuit unpowered in the first training session. This indicates a users’ ability to effectively benefit from the torque provided by the device with a small amount of exposure (Fig. 1, Table 1). These results are in line with previous studies^13,15^, though obtained with a device assisting the hip joint and actuated with a different control strategy. The percentage change of metabolic cost (powered vs unpowered) further increased across sessions so that the participants seemed to be fully adapted at session three, where they displayed a −10.5 ± 4.5% percentage change in metabolic cost (powered vs unpowered). No significant improvement in metabolic cost was reported after session three. Also, it is relevant to note that seven out of eight participants involved in the study registered their highest percentage change of metabolic power after session two, further strengthening the finding that on average two training sessions are likely needed before realizing the maximal metabolic benefit.

While, on average, at least two training sessions were needed to be fully adapted, large variability in percent change of metabolic cost was found across participants; illustrated in the high standard deviation recorded in each session (Fig. 1). It is also relevant to mention that the reduction in metabolic cost was not uniform across the sessions, with some participants exhibiting small increases in energy expenditure when comparing two subsequent sessions. Anthropometric factors could be associated with this individual variability; however this study was not properly powered to assess the influence of height and weight on the effect of training on metabolic cost. Nevertheless, seven out of eight participants reported an additional reduction in the metabolic cost of 4.7% from session one to session five, which almost doubled the metabolic benefit reported during session one. This illustrates the strengthening of evidence of a training effect on metabolic cost with powered exoskeletons. Moreover, the different amount of exposure times necessary for study participants to fully reduce metabolic cost highlights the importance of performing an adequate amount of training before evaluating an exoskeleton’s performance. If users are not fully adapted to a specific device, the outcomes of the measurements could cause misleading interpretations of the true potential for performance augmentation.

This study is also the first to evaluate the retention effect associated with exoskeleton assisted walking. Previous studies investigating neuromuscular adaptations in participants walking with different assistive devices focused on the time needed to learn how to walk with the exoskeleton^7,13,15^ but, to the best of our knowledge, none have investigated the retained metabolic reduction after prolonged periods of non-use. Interestingly, when we retested participants five months after the conclusion of the training study we found that the percentage change in net metabolic power was not significantly different. This suggests that the participants’ retained the adaptations which resulted in reduced metabolic cost while walking with the soft exosuit, and that they are able to quickly regain any metabolic performance increase following up to a five month break in use.

Subjective measurements indicated that the participants perceived a lower level of effort when walking with the soft exosuit powered. This is in agreement with the lower metabolic cost reported in the powered condition, however no correlation was found between perceived exertion and percentage of metabolic cost reduction, nor was there a noticeable effect of training across sessions in the perceived exertion. This finding might indicate that, although the individual perceived a beneficial effect from the soft exosuit, this simple parameter may not be able to be used to monitor adaptation or as a surrogate for metabolic cost. Similar findings have been reported previously when investigating the relationship between subjective evaluations and metabolic cost^11^, indicating that other factors such as comfort or motivation are also likely relevant when it comes to any subjective evaluation.

We investigated the effect of training on stride time, however we found no effect across sessions. We did uncover within-session changes in stride time, with values at *late_pow* closer to those measures at *unpow* compared to *early_pow*. This may be due to initial acclimatization to the exosuit rather than adaptation, as we would expect *late_pow* and *early_pow* to be more closely related if the mechanism was directly linked to the metabolic cost. The magnitude of change in stride time was relatively small within a session (<0.03 s between *early_pow* and *unpow*), possibly indicating that its effect on metabolic cost would be negligible^28^. Nevertheless, future work is needed to better characterize initial changes to exoskeleton assisting gait. In particular, adaptations in metabolic cost could be more closely associated with lower limb musculoskeletal and joint-level changes. We plan to expand the analysis of these variables in our next studies to obtain more insights into the mechanisms responsible of this behavior.

The inability to collect lower limb kinematics and muscle activation, due to the logistical time constraints for participants, is a major limitation to the understanding of the mechanism for continued metabolic reduction with training. Upon completion of the study, we found that respiratory exchange ratio (RER) was initially at 0.97+/−0.06 when the participants were at rest and rose to 1.01+/−0.04 at the end of each session. These values appear elevated as previous work by Goedecke *et al*. has found RER at rest to be 0.812+/−0.05 and RER at 70% of peak power output to be 0.98+/−0.04^29^. All calibration procedures were followed by manufacturer trained personnel with a brand-new measurement unit, oxygen sensor, and sampling lines. After reaching out to the system manufacturer we were informed that our calibrations, while within the acceptable range, showed a relatively low gain for the O2 sensor. This could lead to the artificially elevated RER observed. The observed increase in RER due to testing protocol was only 0.04, lending support that our subjects did not become overly fatigued during each training session. It is important to note that our subjects regularly carry loads of this magnitude at this walking speed for much longer durations. This study design investigated within-session changes in metabolic energy expenditure, therefore any issues with O2 sensor gain are applied equally for within session power calculations and would have limited impact on the overall findings of this research.

Another limitation is the consistent order of powered to unpowered conditions. While this is less likely to influence the change in metabolic cost across sessions, it may influence the percent reduction between powered and unpowered conditions. To investigate the effect of sustained treadmill walking on metabolic cost further, we included additional sessions before the first exosuit training session and after the 5^th^ exosuit training session, where the participants completed the testing protocol without any exosuit. During these sessions, which included only the standard rucksack, our participants had an average increase in metabolic cost of 0.46% and 0.27%, when comparing the middle time period and late time period to the final time period, respectively. These pilot data lend support to the changes in metabolic cost being attributed to the training with the soft exosuit, and not simply due to the individual session walking time or order of our comparisons. It also confirms that our participants were not overly fatigued by the 25 minutes of activity per session.

In conclusion, the present work was the first to examine the metabolic cost adaptations associated with an extended training protocol when walking with a soft exosuit, and also the first investigation on this topic involving a hip exoskeleton of any kind. Furthermore, this work demonstrated that the improvements in metabolic benefit were retained after prolonged non-use. These outcomes are of practical applicability for research groups testing different types of exoskeletons, along with organizations evaluating the performance of current prototypes. The current findings suggest that two 20-minute training sessions are sufficient in order to maximize the reduction in metabolic power provided by a soft exosuit assisting the hip joint. Moreover, participant-specific variability may require some users to undergo additional sessions to become fully adapted to a physical augmentation device. Nevertheless, it remains unclear if improved performance could be achieved by a single 40-minute session, two sessions in the same day, or if different training days are needed to warrant motor consolidation. The lack of changes in metabolic cost between middle and end of training within the same session suggests that multiple days may be necessary. Furthermore, because of the lack of significant reduction within a single training session, it could be speculated that the impact of a shorter session would be equally beneficial. More research is needed to understand adaptation and performance resulting from varied training paradigms, to help reveal underlying musculoskeletal changes resulting in improved performance. These results are important to understand the mechanisms related to learning to walk with a physical augmentation device, which will both improve current performance and assist in designing the next generation device. To this extent, in the future we envision new participant-specific control strategies which could be able to better assist different learning strategies applied by each individual user, allowing a quicker adaptation time and an overall more effective use of the exoskeletons.

## Methods

### Participants

We recruited eight male cadets from the United States Military Academy at West Point (age: 20.6 ± 1.2 yr; height: 1.80 ± 0.09 m; mass: 78.6 ± 9.2 kg) to participate in this study. All participants reported no musculoskeletal injuries or diseases and had no previous experience walking in an exoskeleton of any kind. The study was approved by the Army Research Laboratory Institutional Review Board (Protocol Number ARL 15–039) and each participant provided written informed consent. All methods were performed in accordance with the relevant guidelines and regulations. Utilizing preliminary data from Ding *et al*.^11^, we conducted a power analysis which determined eight participants were needed to detect a 5% reduction in metabolic energy expenditure with a power of 0.90.

### Testing protocol

**Figure 2:**
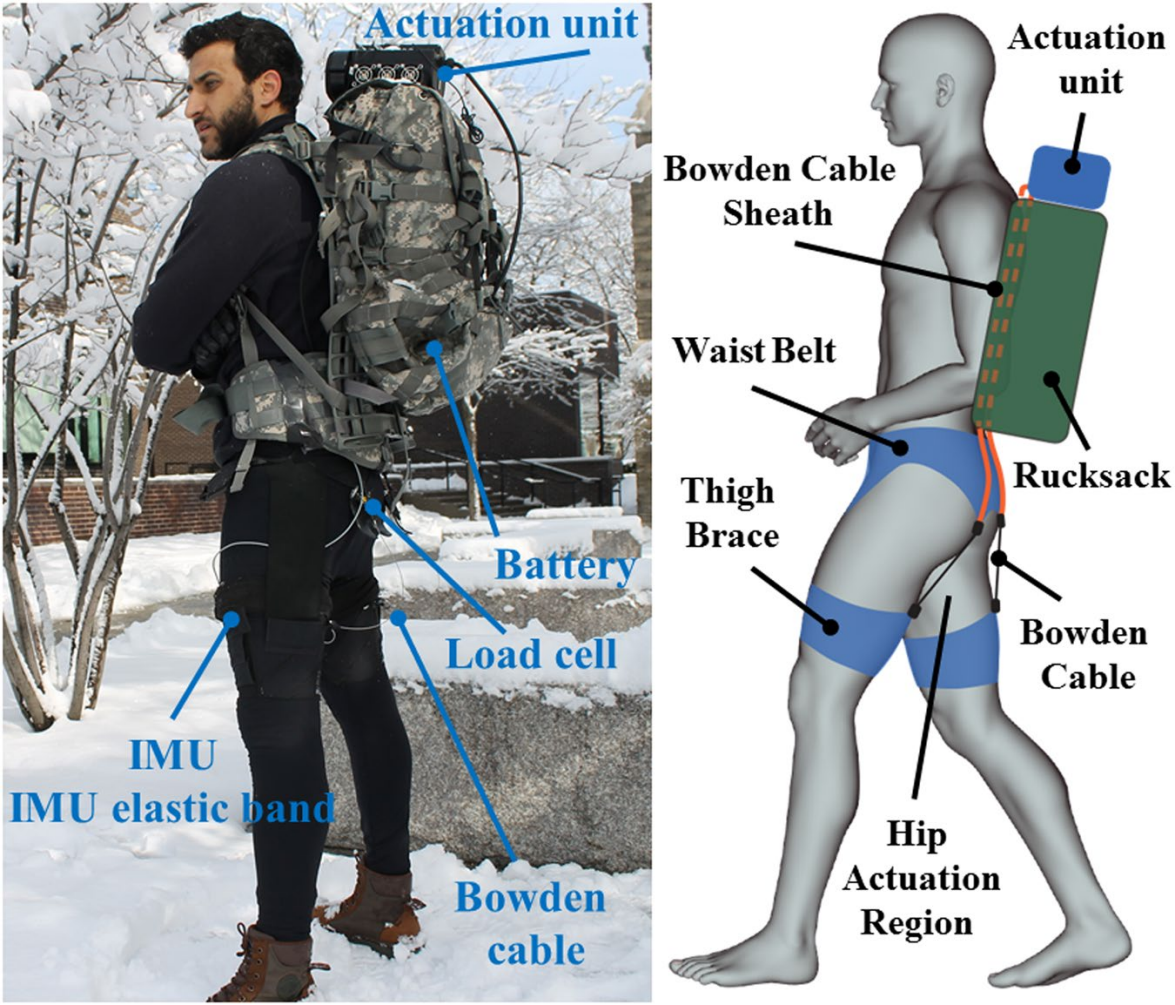
Hip soft exosuit mounted on top of a MOLLE II rucksack, side and back view.

In this study, we investigated how participants learn to use an autonomous mono-articular soft exosuit assisting hip extension (Fig. 2) across multiple training sessions. Each participant completed five training sessions with each session separated by at least 48 hours. The overall training study was completed in no more than 20 days from their first training session (14.3 ± 3.3 days).

Each training session consisted of four minutes of standing, 20 minutes of walking with the exosuit powered, and then five minutes of walking with the exosuit unpowered. Prior to the 20 minutes of powered walking, participants walked for approximately one minute to allow the exosuit’s controller to ramp up the assistive forces to the desired level of 300 N. For data collection and analysis, we segmented the 25 total minutes of walking into four segments: *early_pow* (0–2 minutes), *mid_pow* (9–11 minutes), *late_pow* (18–20 minutes) and *unpow* (23–25 minutes). Between the powered and unpowered walking there was a short break (~30 s) to power off the exosuit. Participants walked at the constant speed of 1.5 m·s^−1^ on an instrumented split-belt treadmill (Bertec, Columbus, OH, USA) while carrying a loaded backpack (20.4 kg) and the soft exosuit actuator, battery and textile components (5.4 kg, Table 4). The exosuit components were carried during the unpowered segment, however the actuation cables were slack and no hip extension or flexion assistance was provided. Five of the eight participants attended three additional sessions (eight total) in order to explore the potential effect of prolonged training time. To understand the effect of retention following the initial adaptation process, five participants were also retested five months after the completion of their last testing session.

**Table 4:** Soft exosuit component mass.

	Components	Mass [g]
Textile	Base layer	222
	Waist belt	435
	Thigh braces	154
	IMU straps	90
Power	Actuation	3100
	Battery pack	1400
Total		5401

### Hip soft exosuit

The device used in this research has been described previously^30^, but a brief description is provided here for completeness. The soft exosuit (Fig. 2) is enabled by Bowden cable driven electromechanical actuation. The textile components of the hip soft exosuit are similar to these used in our prior work^9^ and consisted of a spandex base layer, a waist belt and a waist belt liner, two thigh braces and two inertial measurement unit (IMU) straps. The actuation unit is mounted externally on top of a MOLLE II rucksack (Fig. 2) and the battery is placed on the bottom of the rucksack; the overall mass of all the components of the soft exosuit and its actuation system is 5.4 kg and details are presented in Table 4. The Bowden cable sheath connects to the actuation unit and the bottom of the waist belt, while the inner cable connects to the top center of the thigh brace on the back of each leg. Therefore, when the motor retracts the cable, it delivers a controlled force in parallel to the wearer’s hip extension muscle group, in turn generating an extension torque around the hip joint. Following the motor actuation, the motor feeds out cable during the swing phase of gait and the Bowden cables become slack.

The profile of the force applied to the posterior thigh, which provides the assistive hip extension torque, is controlled by means of an IMU sensor (VectorNav Technologies, Dallas, Texas, US) mounted on the anterior thigh of each leg to detect the maximum hip flexion angle. This gait event is used to trigger the actuation unit, and force-based position control is then used to create the desired hip extension torque as described in^11,31^. Given the variability in hip kinematics, kinetics, suit positioning, and participants’ strides, the controller adjusts the pretension level and the maximum amplitude of the motor position command based on the force profile of the previous gait step. This ensures a consistent force is delivered.

**Figure 3:**
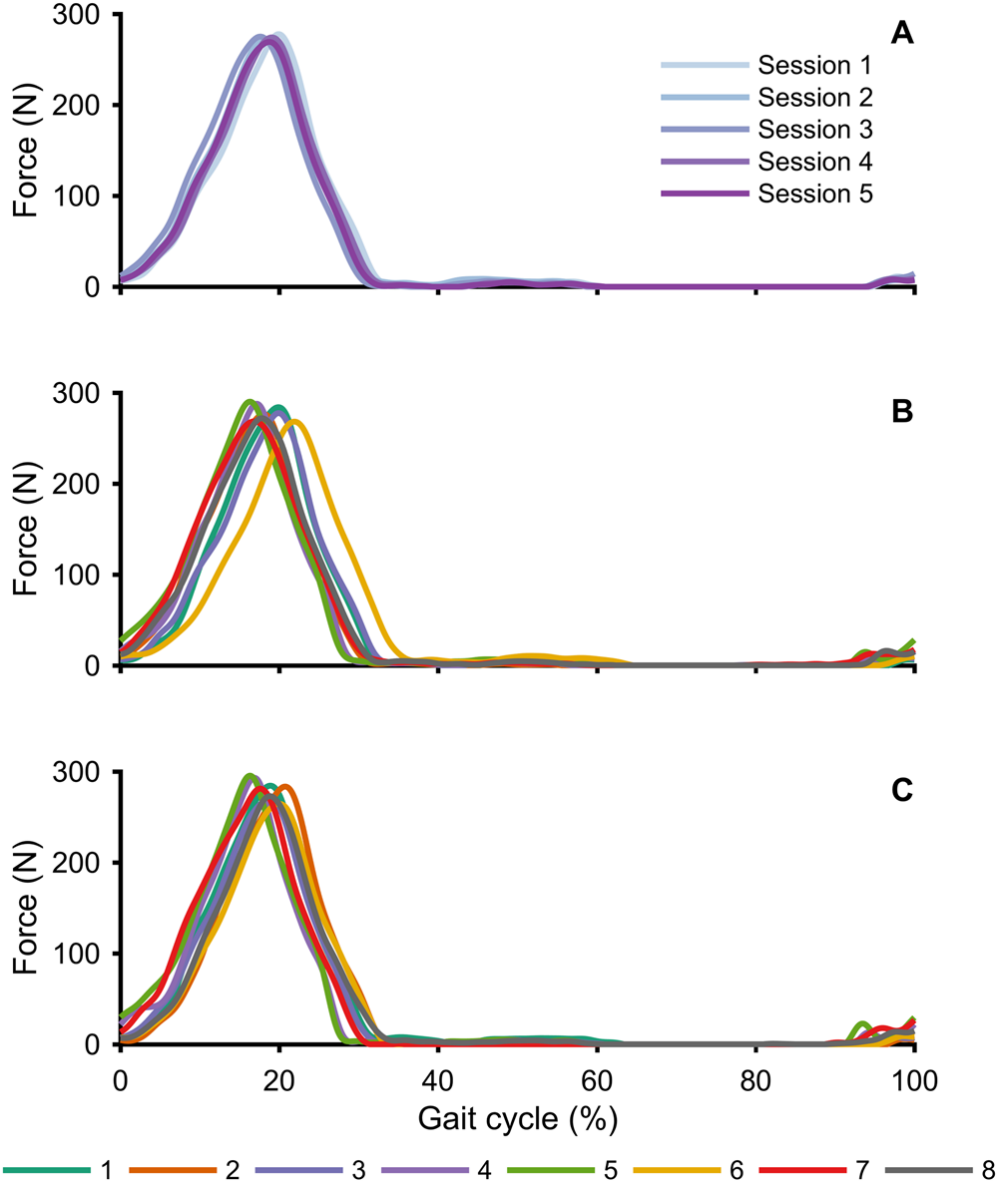
Average assistive force profiles provided at the hip across the gait cycle during min 18–20 (*late_pow*) for: a representative participant during all the testing sessions (**A**); all the participants during session one (**B**) and during session five (**C**).

The soft exosuit delivered constant and symmetric forces across sessions to all participants. Consistency in force profiles was assessed by investigating peak force and three key events of the force traces: onset force timing (percentage of the gait cycle at which the assistive force exceeds the threshold of 5 N), peak force timing (percentage of the gait cycle at which the peak force occurs) and offset force timing (percentage of the gait cycle at which the assistive force goes below the threshold of 5 N) by means of two types of repeated measurement analysis of variance (ANOVA) with Tukey post-hoc correction. One ANOVA analysis investigated within session effects comparing peak force and each of the three force timing events, in three different bouts of walking (*early_pow*, *mid_pow* and *late_pow*), while the other ANOVA analysis investigated peak force and each force timing across session effects. Peak force was consistent across all sessions, with little changes (~5 N) within sessions (Table 5). Analyses of offset and peak force timing reported no significant differences both within session and across sessions for all the participants. A slight shift (<1%) was found in the onset force timing within the sessions, however no differences were reported in this parameter across sessions. Force profiles with respect to the gait cycle are presented for a representative participant during all the testing sessions (Fig. 3A), as well as all the participants during session one (Fig. 3B) and during session five (Fig. 3C).

**Table 5:** Peak force calculated during walking with the soft exosuit powered at minutes 0–2 (*early_pow*) minutes 9–11 (*mid_pow*), at minutes 18–20 (*late_pow*) across the five testing sessions for each participant.

n	Session 1			Session 2			Session 3			Session 4			Session 5		
	*early_pow*	*mid_pow*	*late_pow*	*early_pow*	*mid_pow*	*late_pow*	*early_pow*	*mid_pow*	*late_pow*	*early_pow*	*mid_pow*	*late_pow*	*early_pow*	*mid_pow*	*late_pow*
#1	300 ± 33	306 ± 36	304 ± 35	288 ± 44	308 ± 34	304 ± 33	295 ± 35	302 ± 33	309 ± 31	288 ± 34	308 ± 28	301 ± 34	290 ± 38	308 ± 37	302 ± 36
#2	299 ± 27	300 ± 26	300 ± 28	295 ± 24	295 ± 23	300 ± 19	299 ± 24	298 ± 19	300 ± 24	297 ± 21	299 ± 20	298 ± 20	298 ± 23	297 ± 19	300 ± 23
#3	296 ± 25	299 ± 20	302 ± 22	296 ± 25	300 ± 23	301 ± 22	298 ± 22	297 ± 22	298 ± 21	301 ± 23	299 ± 21	299 ± 18	300 ± 23	303 ± 19	301 ± 20
#4	297 ± 35	294 ± 37	300 ± 34	288 ± 32	298 ± 23	296 ± 27	291 ± 25	293 ± 26	296 ± 29	298 ± 25	298 ± 27	301 ± 27	290 ± 28	300 ± 30	298 ± 38
#5	300 ± 44	296 ± 48	307 ± 35	295 ± 41	299 ± 33	304 ± 36	301 ± 36	304 ± 33	311 ± 32	292 ± 33	304 ± 32	301 ± 30	299 ± 30	305 ± 25	307 ± 26
#6	305 ± 26	302 ± 37	305 ± 34	298 ± 31	294 ± 33	302 ± 26	289 ± 35	273 ± 45	291 ± 37	281 ± 35	280 ± 36	274 ± 41	282 ± 41	298 ± 36	304 ± 33
#7	300 ± 32	302 ± 31	301 ± 28	294 ± 32	298 ± 28	308 ± 31	297 ± 30	302 ± 29	307 ± 27	292 ± 34	305 ± 34	309 ± 33	302 ± 31	302 ± 25	302 ± 32
#8	298 ± 36	306 ± 32	307 ± 29	299 ± 28	305 ± 34	306 ± 38	306 ± 33	307 ± 29	304 ± 31	300 ± 33	306 ± 34	303 ± 27	300 ± 27	299 ± 37	302 ± 28

Data are displayed in Newtons as means ± standard deviation.

### Data collection and analysis

Metabolic cost was assessed with indirect calorimetry by means of a portable gas analysis system (K4b^2^, Cosmed, Roma, Italy). Carbon dioxide and oxygen rate were measured continuously throughout the entire training session (both standing and walking). These measurements were used to calculate metabolic power using a modified Brockway equation^32^. Average metabolic power was calculated for walking at *mid_pow*, *late_pow*, and *unpow* segments using two minutes for each condition. Average metabolic power during the first two minutes of walking (*early_pow*) was not included in the analysis as this did not provide enough time to reach steady state metabolic energy expenditure^32^. Visual inspection of the data showed that participants were at steady state during the other conditions (*mid_pow*, *late_pow*, and *unpow)*. Net metabolic power (normalized to body mass without exoskeleton) during loaded walking was obtained by subtracting the metabolic power obtained during standing from the metabolic power calculated during the walking conditions. Percent change in net metabolic power during powered conditions was calculated using the following equation:

$${\rm{Percent}}\,{\rm{change}}=100\times \frac{{\rm{Powered}}-{\rm{Unpowered}}}{{\rm{Unpowered}}}$$

Participants’ rate of perceived exertion was collected using the Borg scale^33^ at the end of *mid_pow*, *late_pow* and *unpow*. This numerical scale ranges from no exertion quantified as a value of 6 to maximal exertion quantified as 20 and has previously been correlated with heart rate^33^.

Ground reaction forces (GRFs) were collected at 1000 Hz (NI USB-6343, National Instruments Corp., Austin, TX, USA) from the instrumented split-belt treadmill across the four separate segments described above: during powered (*early_pow*, *mid_pow* and *late_pow*) and unpowered (*unpow*) walking. GRFs were low pass filtered using a zero-lag 4^th^ order low pass Butterworth filter with a 6 Hz cut-off frequency (MATLAB, The MathWorks Inc., Natick, MA, USA).

Heel strike events were defined as the point in time when the ground reaction force passed a 50 N threshold, and stride time was calculated as the time between two consecutive ipsilateral heel strike events. Strides containing periods where both feet were on one force plate, due to the participants’ feet crossing the median, were excluded from the analysis.

### Statistical methods

For each variable of interest (net metabolic cost, stride time and perceived exertion), we conducted two separate one-way repeated measurement analyses of variance (ANOVA) with Tukey post-hoc correction; one to examine within session effects and one to examine across sessions effects. This statistical analysis selected was the same that was used by Koller *et al*. in their study relative to adaptations within and across sessions using an ankle exoskeleton^13^. We analyzed three conditions (*mid_pow*, *late_pow* and *unpow)* for net metabolic cost and all four conditions *(early_pow*, *mid_pow*, *late_pow* and *unpow)* for perceived exertion and stride time. Statistical analyses were performed using MATLAB (MATLAB, The MathWorks Inc., Natick, MA, USA) and the significance level was set at p < 0.05 for all analyses.

## Supplementary information

Supplemental material exosuit components

Dataset 1
