# Supplementary material for: Metabolic cost adaptations during training with a soft exosuit assisting the hip joint
Source: Sci Rep. 2019 Jul 5;9:9779. doi: 10.1038/s41598-019-45914-5 (PMC6611879; doi:10.1038/s41598-019-45914-5)
Supplement: Supplementary file 1 — Supplemental material exosuit components [file 41598_2019_45914_MOESM1_ESM.pdf]

Metabolic cost adaptations during training with a soft exosuit assisting the hip joint

Fausto A. Panizzolo[1,2¶], Gregory M. Freisinger[3,4,5¶*], Nikos Karavas[1,2], Asa M. Eckert-Erdheim[1,2], Christopher Siviy[1,2], Andrew Long[1,2], Rebecca A. Zifchock[3], Michael E. LaFiandra[4], Conor J. Walsh[1,2]

[1] John A. Paulson School of Engineering and Applied Sciences, Harvard University, 29 Oxford Street, Cambridge, MA 02138, USA

[2] Wyss Institute for Biologically Inspired Engineering at Harvard, 3 Blackfan Circle, Boston, MA 02115, USA

[3] Department of Civil and Mechanical Engineering, United States Military Academy, 752 Thayer Road, West Point, NY 10996, USA

[4] United States Army Research Laboratory, Aberdeen Proving Ground, 4727 Deer Creek Loop, MD 21005, USA

[5] 75th Innovation Command, United States Army, 10949 Aerospace Ave, Houston, TX 77034, USA

* Corresponding author:
Gregory M. Freisinger
gregory.freisinger@westpoint.edu

[¶] These authors contributed equally to this work.
The views expressed do not reflect the official policy or position of the United States Army, Department of Defense, or the U.S. Government

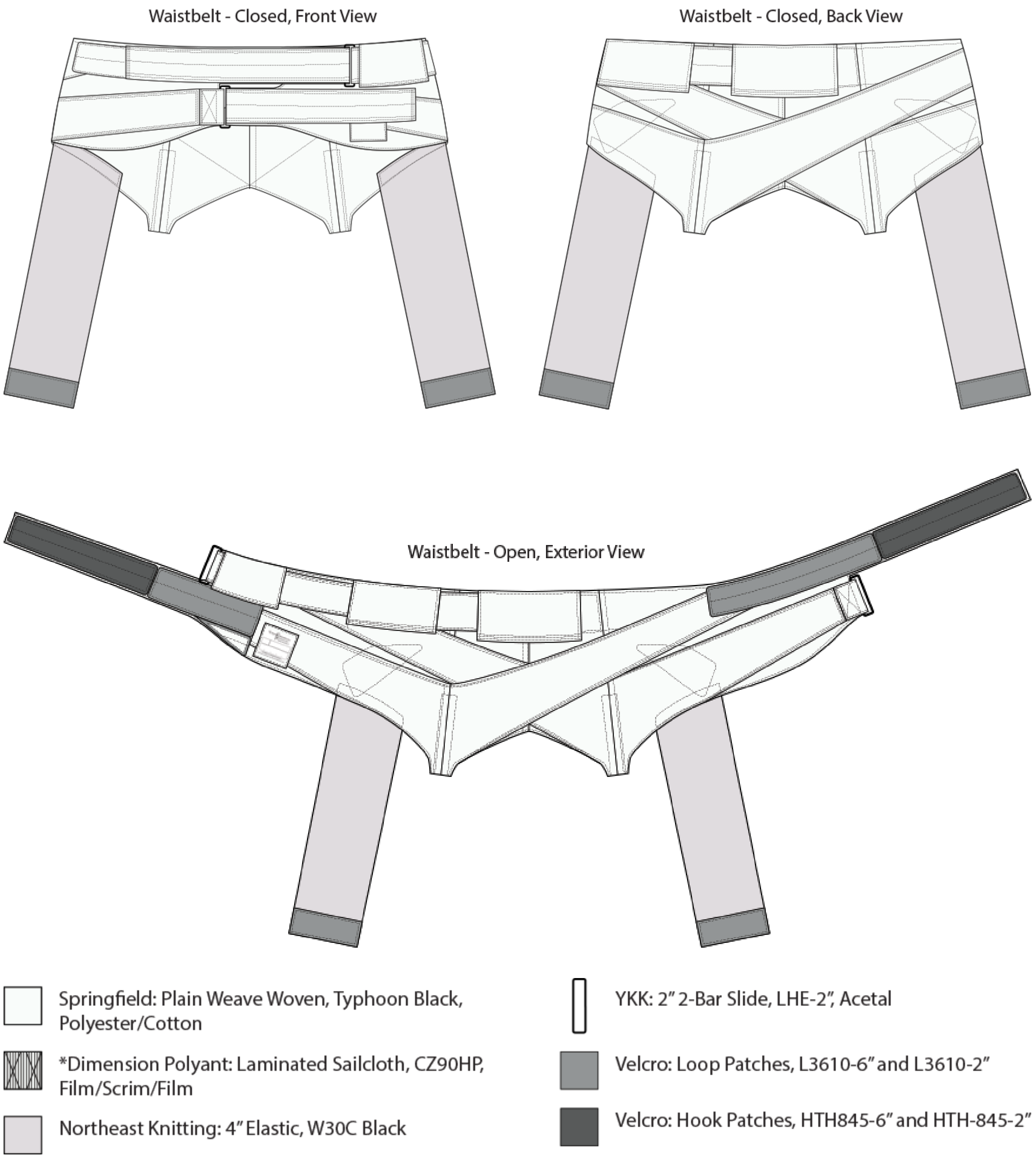

Structure of the waistbelt component. *The waist belt is mainly composed of a plain woven (Typhoon Black, Source: Springfield) sourced to minimize strain. Two different types of Sailcloth Injection Fiber (Source: Dimension Polyant) were used for reinforcement. Hook and Loop Velcro were used for the front closure belts. Additionally, two 2" 2-bar slides were sewn into the closure of each belt. The front closure belts route through these 2-bar slides, allowing the user to adjust the fit and compression of the waistbelt. Attachment points and reinforced load paths can be seen on the back of the waist belt, used to assist hip extension. The 4" elastic strips (Source: Northeast Knitting) at the side seams of each belt are designed to attach to the thigh braces.*

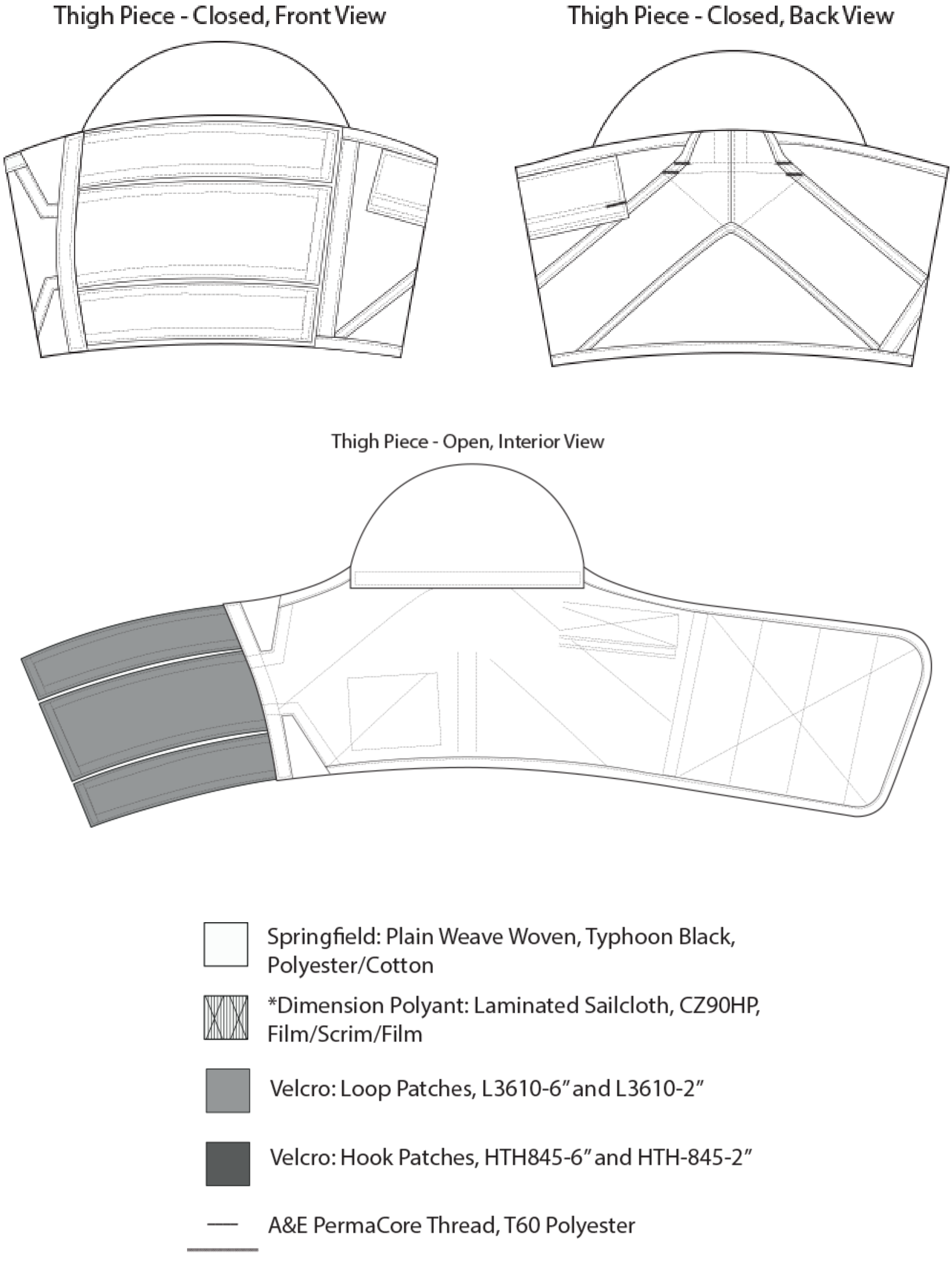

Structure of the thigh piece components. *Like the waist belt, the thigh pieces are mainly composed of a plain woven (Typhoon Black, Source: Springfield) and two different types of Sailcloth Injection Fiber (Source: Dimension Polyant). An attachment point can be seen below the half-moon shape, a guard between the attachment point and the user's center back thigh. The rectangular shape to the left of the attachment point contains hook Velcro patches that receive the 4" elastic strips on the waistbelts (Source: Northeast Knitting). Each thigh brace has two segments of smaller elastic next to its three closure tabs, which allow the thigh brace to conformally curve around conical thighs. The three-tab closure system has loop Velcro, which wraps around the thigh and secures to hook Velcro.*
